# Extract From *Tetrastigma hemsleyanum* Leaf Alleviates *Pseudomonas aeruginosa* Lung Infection: Network Pharmacology Analysis and Experimental Evidence

**DOI:** 10.3389/fphar.2021.587850

**Published:** 2021-07-19

**Authors:** Tian-ling Lou, Tao Ji, Xin Peng, Wei-wei Ji, Li-xia Yuan, Juan Wang, Shi-min Li, Shun Zhang, Qiao-yun Shi

**Affiliations:** ^1^ Zhejiang Pharmaceutical College, Ningbo, China; ^2^ Food and Health Branch, Ningbo Research Institute of Zhejiang University, Ningbo, China; ^3^ Hwa Mei Hospital, University of Chinese Academy of Sciences, Ningbo, China

**Keywords:** *Tetrastigma hemsleyanum*, *Pseudomonas aeruginosa*, Th17 cells, Treg cells, inflammatory immune response

## Abstract

*Tetrastigma hemsleyanum* Diels & Gilg (*T. hemsleyanum*) has attracted much attention due to its ability on pneumonia, bronchitis, and immune-related diseases, while its functional components and underlying mechanism of action on pneumonia have not been fully elucidated. Herein, we used a systematic network pharmacology approach to explore the action mechanism of *T. hemsleyanum* leaf in the treatment of pneumonia. In this study, the results of network pharmacology demonstrated that there were 34 active components and 80 drug–disease targets in *T. hemsleyanum* leaf, which were strongly in connection with signal transduction, inflammatory response, and the oxidation–reduction process. Subsequently, a mouse model of pneumonia induced by *Pseudomonas aeruginosa* (*P. aeruginosa*) was established to validate the predicted results of network pharmacology. In the animal experiments, aqueous extract of *T. hemsleyanum* leaf (EFT) significantly attenuated the histopathological changes of lung tissue in *P. aeruginosa*–induced mice and reduced the number of bacterial colonies in BALFs by 96.84% (*p <* 0.01). Moreover, EFT treatment suppressed the increase of pro-inflammatory cytokines IL-17, IL-6, and TNF-α in lung tissues triggered by *P. aeruginosa*, which led to the increase of Th17 cells (*p <* 0.05). High concentration of EFT treatment (2.0 g/kg) obviously increased the anti-inflammatory cytokine levels, accompanied by the enhancement of Treg proportion in a dose-dependent manner and a notable reversal of transcription factor RORγt expression. These findings demonstrated that network pharmacology was a useful tool for TCM research, and the anti-inflammatory effect of EFT was achieved by maintaining Th17/Treg immune homeostasis and thereby suppressing the inflammatory immune response.

## Introduction

*Pseudomonas aeruginosa* (*P. aeruginosa*), an omnipresent environmental Gram-negative microorganism, is one of the major pathogenic bacteria of hospital-acquired pneumonia (HAP) (Gellatly and Hancock, 2013; Muhammad et al., 2016). HAP chiefly happened in patients with intubation and mechanical ventilation in medical care facilities or intensive care units, while the pathogenic mechanism is inhalation/aspiration of aerosols containing the bacteria, subsequently colonized in the tracheal bronchus (Ali and Ahmad, 2009; Cédric et al., 2013). Similar to other infectious agents, the cargo of *P. aeruginosa* in the upper respiratory region increased and was followed by passing into the lower respiratory tract, which can cause acute pneumonia and fatal bacteremia in critical patients (Ramphal et al., 2008). Characterized by an exceptional mechanism of antibiotic resistance to various antimicrobial agents, nosocomial infection of *P. aeruginosa* has brought an unprecedented therapeutic trouble and was associated with a high risk of fatality (Mesaros et al., 2007). The traditional Chinese medicine (TCM), a unique theoretical system and method of disease diagnosis and treatment, has achieved positive results in the treatment of *P. aeruginosa* lung infection based on clinical practice experience in China (Ma and Wang, 2008; Zhang and Zhu, 2012; Zha, 2018). Therefore, it has become a research hotspot and an alternative source to research and develop new drugs to deeply explore the material basis and mechanism of TCM against *P. aeruginosa* infection.

*Tetrastigma hemsleyanum* Diels & Gilg (*T. hemsleyanum*), known as “San Ye Qing,” belongs to the grape family Vitaceae and is traditionally used to treat pneumonia, hepatitis, children with high fever, and immune-related diseases (Sun et al., 2017; Zhu et al., 2020; Ji et al., 2021). Modern pharmacological studies have shown that *T. hemsleyanum* exhibited significant anti-inflammatory (Ji et al., 2019), antibacterial (Chen et al., 2019), immunomodulatory (Xu et al., 2008), hypoglycemic (Ru et al., 2019a), and antitumor (Peng et al., 2016) effects. Among them, most of the research objects mainly concentrated on the root tubers rather than its leaves. However, as a perennial plant, its root tuber grows slowly and always needs 3–5 years to meet the requirements of commercial medicinal materials (Song et al., 2021). Meanwhile, the leaves of *T. hemsleyanum* were beneficial to health when taken as a functional tea or dietary supplement, such as improving the immune system of the body (Sun et al., 2013). Due to the lack of scientific knowledge and systemic research, the leaves of *T. hemsleyanum* were often discarded, resulting in the low utilization rate of *T. hemsleyanum* resources and insufficient supply of medicinal materials in market. In the recent years, numerous studies have reported that leaves of *T. hemsleyanum* also had high biological activities which were attributed to its many chemical components, such as flavonoids, amino acids, polysaccharides (Ru et al., 2019b), terpenes, cardiac glycosides, and steroids (Sun et al., 2017; Sun et al, 2015). However, the effectiveness and mechanism remained to be further tapped due to its complicated constituents and the lack of suitable methods.

As a promising research, network pharmacology is in accordance with the theory that TCM emphasizes the diagnosis and treatment of diseases from a systematic perspective, and provides a holistic understanding of the mechanisms of multi-ingredient medicine and the synergistic effects of their compounds. Therefore, network pharmacology can be used to understand the scientific basis of TCM at the molecular level and from a system perspective. To date, it has been successfully applied to excavate the multi-target and multi-pathway effect of TCM in various diseases (Guo et al., 2019; Ji W. et al., 2019; Zhang et al., 2019; Lu et al., 2020; Sun et al., 2020). In the present study, we explore the component–target–pathway relationship of *T. hemsleyanum* on pneumonia using the network pharmacology method. Then we established a mouse model of pneumonia induced by *P. aeruginosa* to validate the curative effects and mechanisms of *T. hemsleyanum* leaf on the lung injury. The detailed technical strategy of the present study is exhibited in Supplementary Figure S1.

## Materials and Methods

### Screening of Chemical Compositions in *T. hemsleyanum*

The chemical composition of *T. hemsleyanum* leaf was screened from domestic and overseas literatures published in known databases using the relevant keywords, such as “San Ye Qing,” “golden wire hanging gourd,” “golden bell,” and “golden wire hanging potato.” After eliminating identical data, a total of 94 compounds were obtained, including 54 flavonoids, 24 phenolic acids, 3 anthraquinones, 13 terpenes, and steroids. The molecular structure of the above compounds was mapped in Chembiodraw Ultra1 software, and the PubChem database (https://pubchem.ncbi.nlm.nih.gov) was used to confirm its molecular structure, and then it was saved in MDL molfile format.

### Screening of Compound-Related Targets and Pneumonia-Related Targets

SwissTargetPrediction database (http://www.swisstargetprediction.ch/) and UniProt database (https://www.uniprot.org/) were used to identify the relevant targets of the compound based on chemical similarities and pharmacophore models (Gfeller et al., 2014). Specifically, the structural formula of the chemical components was imported into SwissTargetPrediction database for target prediction, and the selected species was “*Homo sapiens.*” In SwissTargetPrediction database, the probability value is employed to rank the targets and evaluate the accuracy of the predictions. In our present study, we selected targets with probability value ≥0.5. The standard gene names of target proteins were acquired from UniProtKB search function in UniProt database by defining the species as “*Homo sapiens.*” Finally, the target gene was merged and the same gene was deleted to keep the only item, and the results were the targets of the active component of *T. hemsleyanum*.

The acknowledged pneumonia-related targets were collected from DigSee (http://210.107.182.61/geneSearch/), GeneCards (https://www.genecards.org/), and OMIM (http://www.ncbi.nlm.nih.gov/omim) databases. The standard gene names of pneumonia-related targets were also acquired from UniprotKB database. Finally, the targets of the three databases were merged, and the duplicate targets were deleted to keep the item unique; the results were the pneumonia-related target.

The targets of active components in *T. hemsleyanum* and pneumonia-related targets were uploaded to the online Venn diagram package (http://bioinfogp.cnb.csic.es/tools/venny/index.html) for mapping; that is, the intersection of the two was taken to obtain the potential target of *T. hemsleyanum* in the treatment of pneumonia.

### Construction of Target Protein Interaction Network and Screening of Key Targets

The protein–protein interactions (PPI) of each target were generated by the String database (http://string-db.org/), which provides known and predicted interaction information on the basis of systematic co-expression analysis, detection of shared selective signals across genomes, and automated text-mining of the scientific literature (Szklarczyk et al., 2017; Zeng et al., 2019). The species was set as “*Homo sapiens*,” and the interactions with a high interaction score ≥0.900 were selected in the present study. As an available tool for analyzing and visualizing biological networks, the herb–compound–target network of *T. hemsleyanum*, pneumonia–target network, and the network of *T. hemsleyanum* potential target–pneumonia target were constructed using Cytoscape software (version 3.6.1). The central network analysis was performed by the topological method. Degree centrality, betweenness centrality, and closeness centrality, three topological parameters which indicated the importance of network node topology in the networks, were also analyzed using Cytoscape software.

### GO and KEGG Pathway Enrichment Analyses

The gene ontology (GO) enrichment analysis and Kyoto Encyclopedia of Genes and Genomes (KEGG) pathway were performed using the online functional annotation and enrichment tool DAVID (https://david.ncifcrf.gov/). GO terms were associated with biological processes (BP), cellular components (CC), and molecular functions (MF). GO terms and KEGG pathways with *p* value < 0.01 were considered to be statistically significant. Pathways with *p*-value less than 0.01 and target numbers greater than three were selected. Finally, Cytoscape3.6.0 was used to build the drug–compound–target–disease network.

### Sample Preparation

The fresh leaves of *T. hemsleyanum* were collected on November 1, 2018 from planting base of Ningbo Shengwang Biological Technology Co. Ltd. Its biological characteristics were identified by the corresponding author. After collection, the leaves were dried at 40°C, numbered S20181101, and deposited in the specimen room of the Institute of Medical Biotechnology, Zhejiang pharmaceutical College. The aqueous extract from *T. hemsleyanum* leaf (EFT) was prepared in our laboratory. Briefly, 20 g of the powdered *T. hemsleyanum* leaf was weighed and soaked in 200 ml ultrapure water for 30 min, and then ultrasonically extracted for 45 min at 40°C; the ultrasonic extraction was performed using water refluxing for 60 min at 55°C. The refluxed extraction solution was centrifuged at 3,000 rpm for 10 min to collect the supernatant, and the insoluble residue was repeated for two times as described above. The total extract was concentrated to 100 ml using a rotary vacuum evaporator (N-1200AT, Eyela, Japan) at 50°C. The highest concentration of the extraction is 0.2 g/ml, the stock solution was diluted to 0.1 g/ml and 0.05 g/ml with ultrapure water, and the latter was centrifuged at 3,000 rpm for 10 min and filtered with a 0.45-μm filter paper and preserved at -20°C for further use.

### Phytochemical Analysis of EFT

The chemical profile analysis of EFT was conducted by UPLC. Briefly, 10 μl of EFT and standards were, respectively, injected into the UPLC system equipped with a DAD detector (Diane Technologies, United States), and separated on Agilent SB-C18 (4.6 × 250 mm, 5 μm) column at 30°C. The mobile phase was acetonitrile (solvent A) and 0.1% (v/v) phosphoric acid in H_2_O (solvent B). The gradient elution program was performed as follows: 0–30 min, 10–25% A; 30–40 min, 25–95% A; 40–45 min, 95% A; and 45–60 min, 95–100% A. The flow rate was 0.8 ml/min, and the monitoring wavelength was set at 320 nm. The compounds of EFT were identified and quantified based on their spectra and standards. All experiments were repeated three times.

### Experimental Animal and Drugs

One hundred thirty-two ICR mice (8 weeks of age, male), weighing 22 ± 2 g, were purchased from Zhejiang Laboratory Animal Center (Zhejiang, China). All mice were housed at 25 ± 2°C with an alternating 12 h light and dark cycle. Mice were given free access to standard laboratory food and water. All animal experiments were approved by the Ethics Committee of Zhejiang Pharmaceutical College. The mice were randomly divided into six groups of 22 mice each as follows: sham control group; *P. aeruginosa*–infected model group; low, medium, and high EFT treatment groups (low: 0.5 g/kg; medium: 1 g/kg; and high: 2 g/kg); and amikacin (25 mg/kg) treatment. Mice in the sham group were intratracheally injected with aseptic PBS solution. Mice in other groups were intratracheally injected with a dose of 5 × 10^6^ of the planktonic *P. aeruginosa* strain PAO1 (ATCC 27853) as described by Simone (Simone et al., 2014; Simone et al., 2016), which was purchased from Guangdong Food Microbiological Safety Engineering Technology Research and Development Center. Then 12 mice from each group were randomly selected for subsequent administration. After 12 h, mice in the sham and model groups were administered with distilled water (0.2 ml/10 g, ig, twice daily). Mice in the amikacin group were administered with amikacin (25 mg/kg, ig, twice daily), and mice in EFT groups were administered with EFT (0.5, 1.0, and 2.0 g/kg, ig, twice daily). All administrations were conducted for seven consecutive days. The mice were kept fasted for 12 h after the last administration and sacrificed on the seventh day. Blood sample and lung tissue were collected.

### Histological Analysis

The upper page of right lung tissue was isolated from the mice and inflated with 10% formalin for 10 min, and then ligated and removed. Inflated lungs were fixed for 24 h and then embedded in paraffin. Tissue sections (5 μm thick) were stained with hematoxylin–eosin (HE) and observed under an Olympus microscope (Tokyo, Japan). Continuous midsagittal sections were taken for morphological and histological analyses, including the pathological changes of the alveolar structure in mice, pulmonary necrosis, and inflammatory in lung tissue.

### Inflammatory Cytokines Measurement

Blood samples of the orbital venous plexus and BALFs were collected under mild pentobarbital anesthesia, centrifuged at 3,000 rpm for 10 min at room temperature, and then were stored at -80°C for enzyme-linked immunosorbent assay (ELISA). According to the manufacturer’s instructions, ELISA kits were used to detect the serum levels of interleukin (IL)-17, IL-6, IL-10, transforming growth factor (TGF)-β1, and tumor necrosis factor (TNF)-α in blood samples and BALFs.

### Bacteriological Examination *In Vivo*

BALFs were collected by lavaging the upper page of left lung tissue with 2 × 0.5 ml saline as previously reported (Hu et al., 2018). In short, the trachea was exposed again and 0.5 ml sterile saline was slowly injected into the lungs and carefully retrieved. Bronchoalveolar lavage was performed twice for each mouse. 100 μL BALFs were evenly distributed on the nutritional agar medium for bacterial culturing. The number of bacterial colonies was observed and calculated 24 h later. The numbers of bacteria were calculated by measuring the absorbance of 600 nm wavelength (OD600 = 0.5 corresponded to a bacterial concentration of 3 × 10^8^ cfu/ml).

### Real-Time PCR Analysis

Total RNA was extracted from the second page of left lung tissue with Trizol reagent (Life Technologies, Carlsbad, CA, United States) according to the manufacturer’s instructions. For reverse transcription, 2.0 μg of total RNA was reverse-transcribed using a HiScript™ Q RT SuperMix for qPCR (+gDNA wiper). Quantitative real-time PCR was performed with SYBR Green Ⅰ using a Thermal Cycler Dice™ Real Time System (Takara, Japan) according to the manufacturer’s instructions. A relative mRNA level was calculated by the 2^−ΔΔCt^, and glyceraldehyde-3-phosphate dehydrogenase (GAPDH) primers were used to apply to all samples for normalization. Three technical replicates were performed for each sample. The primer sequences used in this study were as follows:

Foxp3: 5′-ACC​CAG​GAA​AGA​CAG​CAA​CC-3′ (forward)

5′-CTC​GAA​GAC​CTT​CTC​ACA​ACC​A (reverse);

RORγt: 5′-CCA​TTG​ACC​GAA​CCA​GCC-3′ (forward)

5′-TCT​GCT​TCT​TGG​ACA​TTC​GG-3′ (reverse);

GAPDH: 5′-TGT​GTC​CGT​CGT​GGA​TCT​GA-3′ (forward)

5′-TCT​GCT​TCT​TGG​ACA​TTC​GG-3′ (reverse).

### Flow Cytometry Analysis

As described previously (Vermaelen et al., 2001), lung single-cell suspensions were prepared from the second page of the right lung. Simply, the lung was thoroughly minced, digested, passed through a 70-μm cell strainer, washed twice with cold PBS at 300×g for 10 min at 4°C, and resuspended with PBS. The mononuclear cells were separated from the lung single-cell suspension by Ficoll-Hypaque gradient centrifugation (Pharmacia, Uppsala, Sweden), washed twice with PBS, and frozen until labeled. The expressions of T-cell markers were determined by flow cytometry after staining with fluorescence-labeled antibody APC-conjugated anti-mouse CD25 and FITC-conjugated anti-mouse CD4 or isotypes (eBioscience, United States) for 20 min at 4°C. These cells were washed twice, fixed, permeabilized, and then stained with PE-conjugated anti-mouse Foxp3 for analysis of Treg subpopulations. Splenocytes were stimulated with 50 μl phorbol 12-myristate 13-acetate (PMA) (1 μg/ml, BioVision, Mountain View, CA, United States), 40 μl ionomycin (50 μg/ml, Enzo Life Sciences, Farmingdale, NY, United States), and 20 μl monensin (0.1 mg/ml, eBscience, San Diego, CA, United States) for 4 h and were stained with FITC-conjugated anti-mouse CD4 and PE-conjugated anti-mouse IL-17 antibodies in order to analyze Th17 cells. Flow cytometry was carried out using a FACS Canto II flow cytometer (BD, United States). The percentages of Th17 cells and Treg cells were analyzed.

### Statistical Analysis

Quantitative data were presented as mean ± standard deviation (SD). The differences among the groups were assessed by one-way ANOVA. Correlation coefficients were calculated using Pearson’s rank correlation test. *p <* 0.05 was considered statistically significant. All statistical analyses were performed using SPSS statistical software version 13.0.

## Results

### Prediction of Active Components and Potential Targets of *T. hemsleyanum* in the Treatment of Pneumonia Based on Network Pharmacology

**FIGURE 1:**
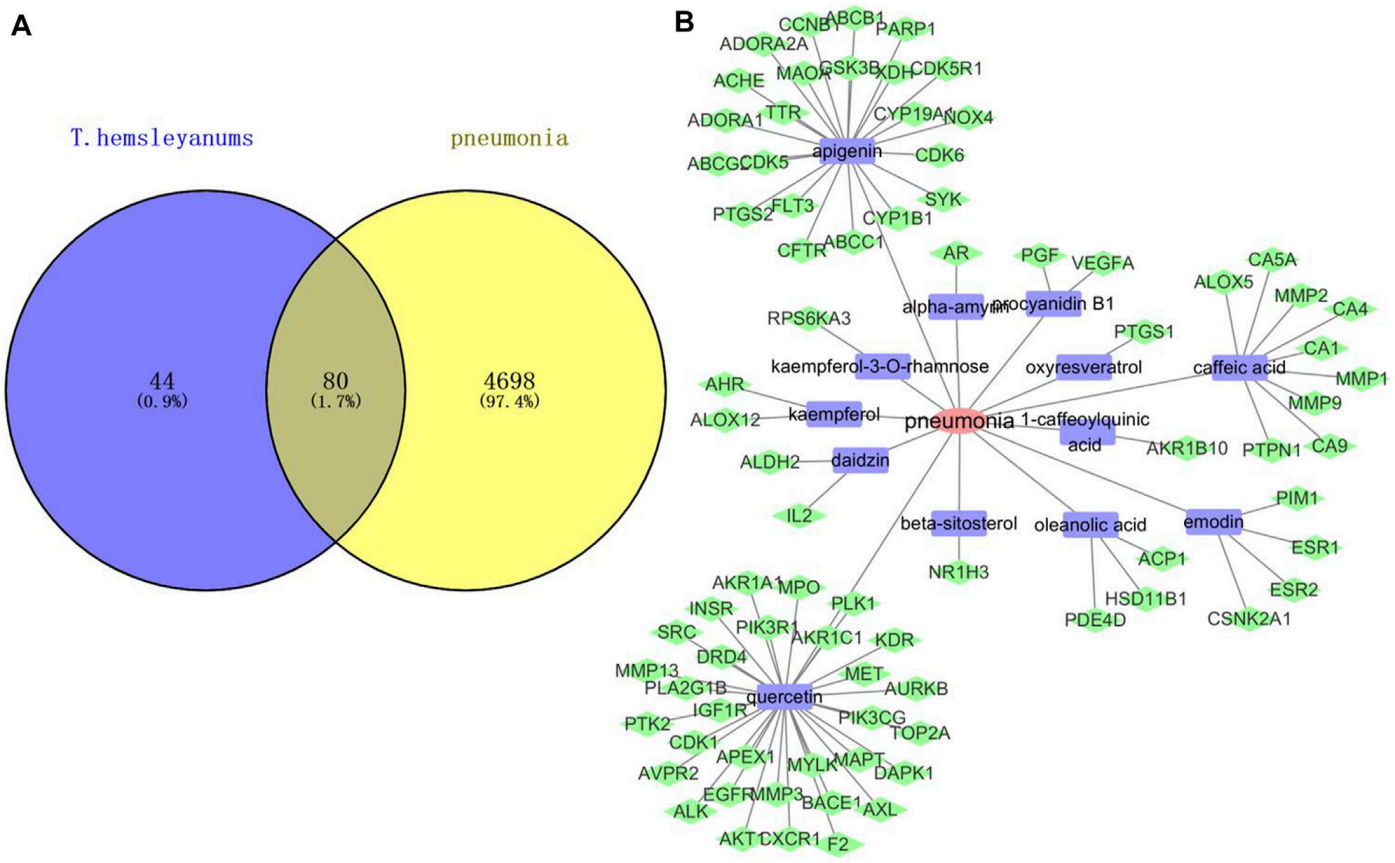
Network analysis of disease and compound targets. **(A)** Venn diagram of *T. hemsleyanum* targets and pneumonia targets; **(B)** the disease–compound–target network of *T. hemsleyanum* leaf. Green represented drug–disease intersection proteins, red represented pneumonia, and blue represented active compounds.

A total of 34 effective components of *T. hemsleyanum* leaf were collected in the present study according to the screening conditions, and 124 compound-related targets with 324 frequencies were found after eliminating the duplicates. These findings revealed that the effectiveness of *T. hemsleyanum* leaf against complex diseases depended on the synergy between multiple compounds and their targets. Although the target number of each component was varied, there was significant overlap among the 34 components. As for disease target identification, we retrieved collected 4,779 pneumonia-related targets from the OMIM, Gene Cards, and DigSee databases after eliminating overlapping targets. Moreover, a total of 80 genes were identified with Venn diagrams, which were related to both *T. hemsleyanum* leaf and pneumonia, and the result is exhibited in Figure 1A. After excluding overlapping compounds, a total of 13 compounds in *T. hemsleyanum* leaf were inextricably linked with 80 pneumonia-related targets. In order to clarify the relationship among the disease, active compounds, and potential targets, a disease–compound–target network was constructed with 13 compounds and their corresponding targets, and is presented in Figure 1B. The network consisted of 94 nodes, including 1 disease, 13 bioactive compounds (Supplementary Figure S2), and 80 targets. The red, blue, and green nodes represented disease, bioactive compounds, and targets, respectively. There were 93 edges in the network, and the edges represented the interactions among them. In addition, quercetin, apigenin, and caffeic acid were predicted as the crucial active compounds through topological analysis, suggesting their critical roles in *T. hemsleyanum* leaf for pneumonia treatment.

### Construction and Analysis of PPI Network

**FIGURE 2:**
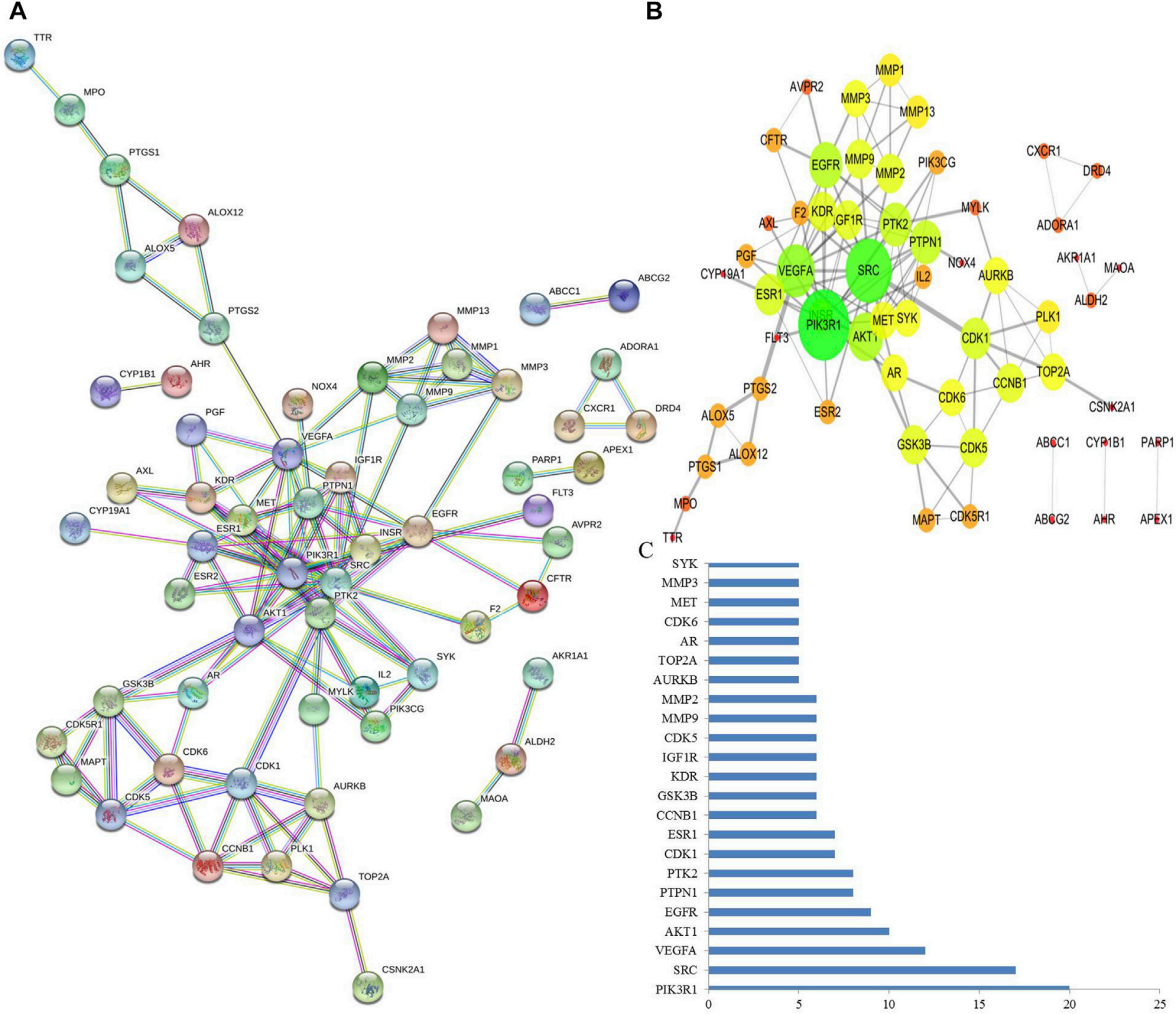
Analysis of PPI networks for protein–protein interactions. **(A)** Nodes in the network indicated proteins. The empty nodes indicated the unknown 3D structure proteins, and the filled nodes indicated known or predicted 3D structure proteins. The edges indicated protein–protein associations. Different colored edges indicated different interactions. **(B)** The protein–protein interaction was mimicked by Cytoscape software. The size and color of the node indicated the degree value of node, and the thickness of edge represents the combined score. **(C)** PIK3R1 is probably the most pivotal hub target that was defined as the number of protein–protein associations more than five.

PPI networks were constructed through a STRING database to explore the potential protein–protein interactions among the pneumonia targets (Figure 2A) and *T. hemsleyanum*, and Cytoscape was used to carry out the visual composition. The nodes and edges in the network indicated target proteins and protein–protein associations. After eliminating disconnected nodes, there were 60 nodes and 128 edges in the network (Figure 2B). The size and color of the node indicated the degree value of target protein, the size of the node was larger, and corresponding degree value was greater. The color changes from red to green, and the corresponding degree value was greater. The thickness of the edge represented the reaction connection score: the thicker the edge, the higher the score. The average node degree and the average local clustering coefficient were 3.2 and 0.512, respectively. As the key node to evaluate the essence of the whole network, hub target was identified by a number of interactions. As shown in Figure 2C, PIK3R1 was the most pivotal hub target in the network. It was a subunit alpha of phosphatidylinositol 3-kinase regulatory, and could bind to activated (phosphorylated) protein-Tyr kinases through its SH2 domain.

### Predicting Functional Enrichment Analysis for *T. hemsleyanum*

**FIGURE 3:**
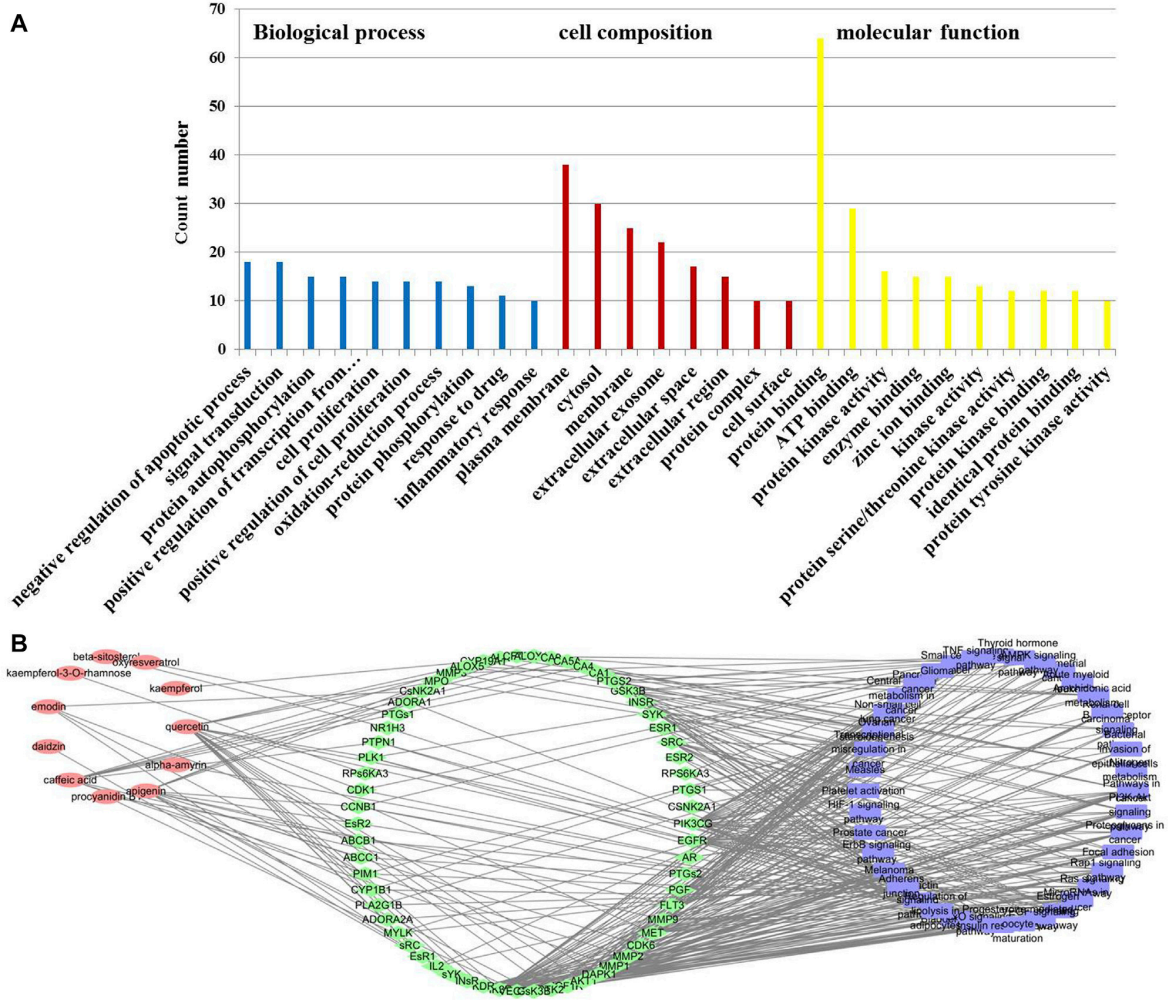
The potential target–pneumonia target network and analysis of *T. hemsleyanum* leaf. **(A)** GO enrichment analysis for 80 drug–disease targets. **(B)** The active component–target–KEGG pathway of *T. hemsleyanum* leaf. Green indicates drug–disease intersection proteins, red indicates active compounds, and blue indicates the pathway.

To elucidate the mechanism of *T. hemsleyanum* leaf in the treatment of pneumonia, the 80 drug–disease targets were put into DAVID database to enrich the pathways. GO analysis demonstrated that a total of 130 records were obtained (*p* < 0.01), including 83 for BP, 13 for CC, and 34 for MF. The results showed that the drug–disease targets were mainly relevant to protein kinase activity, protein phosphorylation, signal transduction, ATP binding, oxidation–reduction process, and inflammatory response (Figure 3A). Moreover, the KEGG enrichment analysis showed that a total of 73 pathways were obtained, of which 40 pathways were statistically significant (*p* < 0.01), and many target genes were strongly in connection with the Rap1 signaling pathway, PI3K–Akt signaling pathway, and Ras signaling pathway. The active component–target–pathway network of *T. hemsleyanum* leaf was visualized by using the software of Cytoscape and depicted in Figure 3B. As shown in Figure 3B, the same active ingredient corresponded to multiple targets, and multiple targets corresponded to the same pathway, which reflected the multi-component, multi-target, and multi-pathway action characteristics of *T. hemsleyanum*.

### Identification of Bioactive Components in EFT

The HPLC chromatograms of reference standards and EFT sample were exhibited in Supplementary Figure S3, and approximately seven chromatographic peaks were identified as the phytochemical profile of EFT, including quercetin-3-O-rutinoside, kaempferol-3-O-rutinoside, kaempferol-3-glucoside, resveratrol, quercetin, kaempferol, and β-sitosterol. As shown in Table 1, β-sitosterol was the most abundant component, followed by quercetin-3-O-rutinoside and resveratrol.

**TABLE 1:** Contents of each bioactive component in EFT (*n* = 3).

Bioactive components	Contents (μg/g)
Quercetin-3-O-rutinoside	44.23 ± 3.46
Kaempferol-3-O-rutinoside	3.60 ± 0.54
Kaempferol-3-glucoside	12.83 ± 1.08
Resveratrol	20.92 ± 2.44
Quercetin	2.43 ± 0.18
Kaempferol	12.71 ± 1.32
β-sitosterol	435.08 ± 28.62

### The Effect of EFT on the Change of Histopathology

**FIGURE 4:**
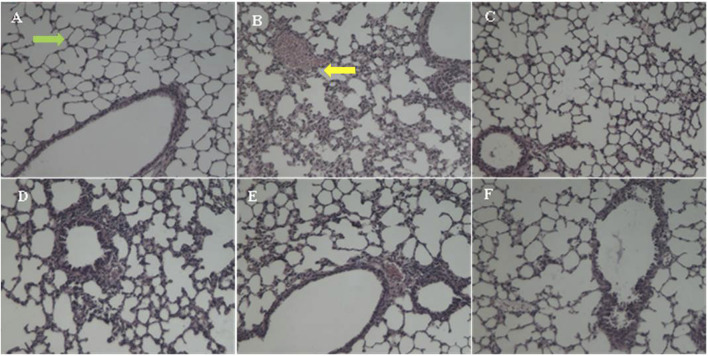
The HE-stained change of lung histopathology. **(A)** The sham group with intact alveoli (green arrow), **(B)** the model group with massive inflammatory infiltrates (yellow arrow), **(C)** the amikacin group, **(D)** the EFT (0.5 g/kg) group, **(E)** the EFT (1.0 g/kg) group, and **(F)** the EFT (2.0 g/kg) group.

The pathological changes of the lung tissue were observed and shown in Figure 4. The lung sections of the sham control group mice showed normal alveolar structure with intact alveoli and no exudate (Figure 4A). In contrast, the lung sections of the model group mice had severe lung damage after infected by *P. aeruginosa* (Figure 4B). Specifically, there was a large number of inflammatory cells accumulation and infiltration in alveoli. Moreover, the amount of alveolar macrophages infiltrating the alveolar septa was increased along with the increase of the quantity of necrosis and inflammatory infiltrates in lung. Remarkably, the lung histology damage of mice was significantly alleviated after taking EFT or amikacin, considering the disappearance of the exudates and the decrease of inflammatory cell infiltrates of bronchi and perivascular edema (Figures 4C–F).

### The Effect of EFT on the Change of Bacterial Colony in BALFs

**FIGURE 5:**
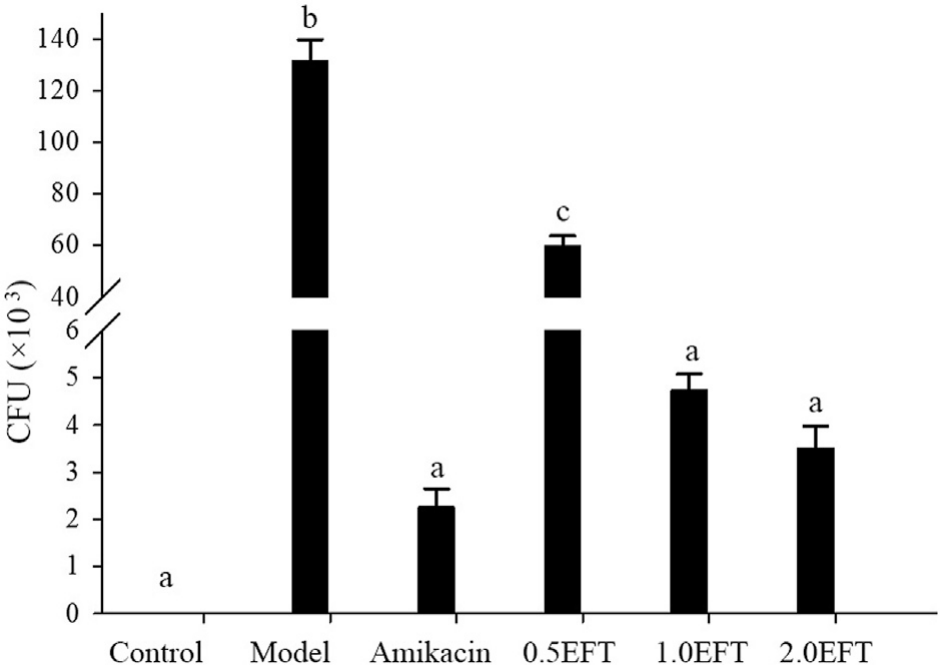
Colony counts in BALFs of mice in each group were collected for culturing, and the number of bacterial colonies was counted 24 h later (*n* = 12). Different English letters in the same column represented a significant difference through pair-wise comparison by the LSD multiple comparison test (*p <* 0.01).

To confirm the successful establishment of the *P. aeruginosa* lung infection mice models and the bactericidal efficacy of EFT *in vivo*, the bacterial burdens in harvested BALFs were estimated. As shown in Figure 5, the bacterial burden in the model group was significantly higher than that in the sham control group, and reached up to 131.66 ± 8.33 × 10^3^ CFU/ml (*p* < 0.01); the bacterial burdens were remarkably decreased in the EFT and amikacin groups compared with the model group (*p* < 0.01), and the bacterial load in the model group was more than 30 times that in the EFT (2.0 g/kg) group. Moreover, the antibacterial effect of EFT was in a dose-dependent manner considering that the bacterial burden in the EFT (2.0 g/kg) group was only 6.4% of that in the EFT (0.5 g/kg) group (*p* < 0.01).

### The Effect of EFT on Inflammatory Cytokine in Serum and BALFs

**FIGURE 6:**
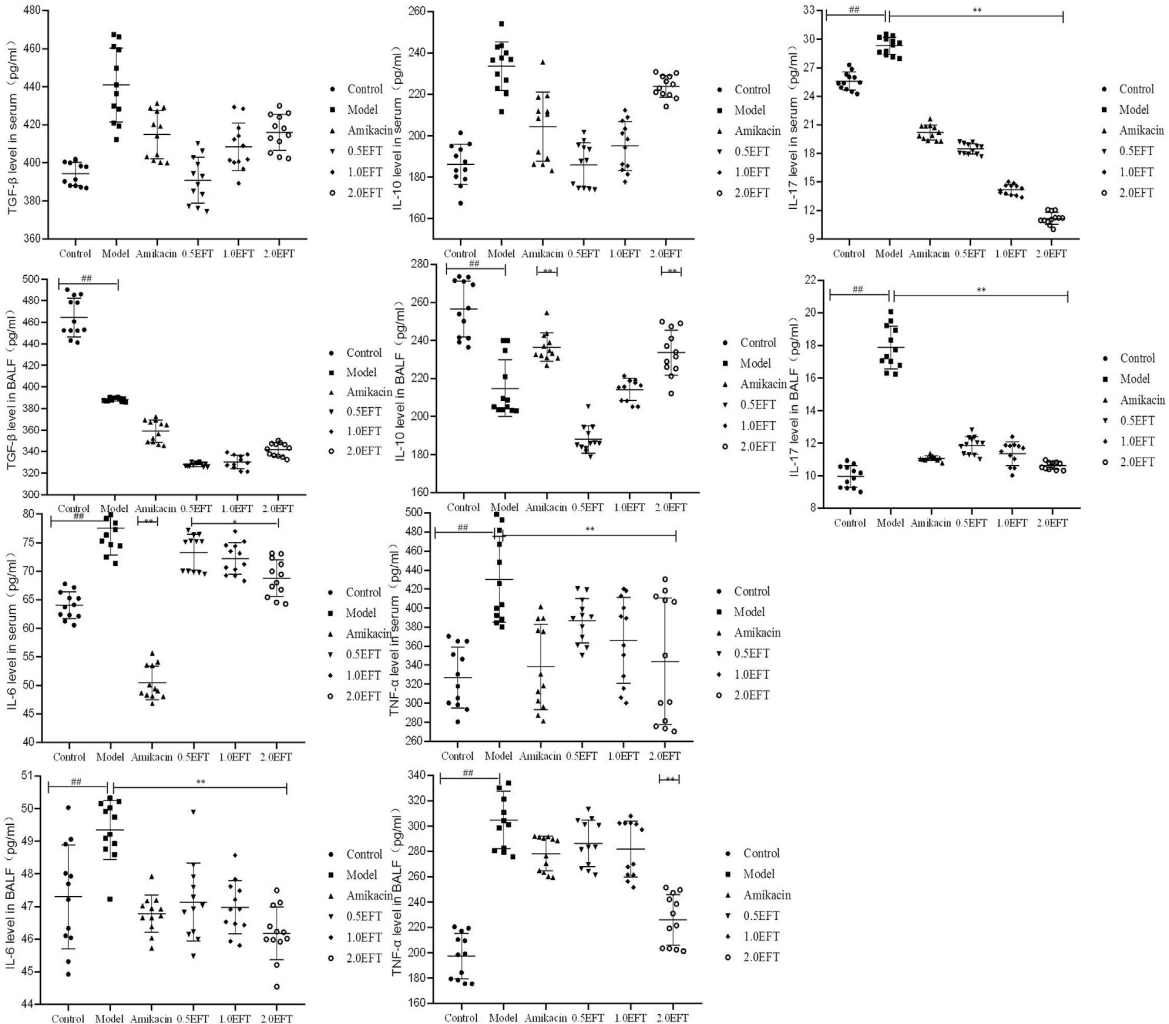
Effects of EFT on inflammatory cytokine in serum and BALFs of each mice group. All values are expressed as mean ± SD (*n* = 12). #*p* < 0.05; ##*p* < 0.01 compared with the control group, *p < 0.05; **p < 0.01 compared with the model group.

As indicated in the network pharmacology analysis, the underlying pathway of *T. hemsleyanum* against pneumonia was remarkably associated with the inflammatory response. Therefore, the levels of pro-inflammatory (IL-17, IL-6, and TNF-α) and anti-inflammatory cytokines (TGF-β1 and IL-10) in serum and BALFs were determined by ELISA. There were no significant differences in the levels of TGF-β and IL-10 in serum between the groups. But the levels of TGF-β and IL-10 in BALFs decreased in the model group compared to the control group (Figure 6). The levels of IL-10 in the amikacin and EFT groups (2.0 g/kg) were obviously increased by 17.6 and 16.9%, respectively, compared with the model group (*p* < 0.05). In contrast, the levels of IL-17, IL-6, and TNF-α in serum and BALFs of the EFT group were lower than those in the model group. The downregulation of IL-17 in serum of EFT groups (0.5, 1.0, and 2.0 g/kg) was 36.82, 51.68, and 61.95%, respectively. Meanwhile, its production in BALFs of mice treated with 0.5, 1.0, and 2.0 g/kg EFT was decreased by 33.47, 36.32, and 40.41%, respectively. The reduction ratios of IL-6 in serum of three EFT groups were 5.25, 6.50, and 11.16%, and those of TNF-α were 10.36, 15.70, and 20.09%, respectively. Meanwhile, the changes of IL-17 in BALFs of EFT groups (0.5, 1.0, and 2.0 g/kg) were accompanied by the attenuation of IL-6 and TNF-α; the former were 4.97, 5.26, and 7.18%, and the latter were 6.03, 7.47, and 25.98%, respectively.

### The Effect of EFT on Treg and Th17 Cells

**FIGURE 7:**
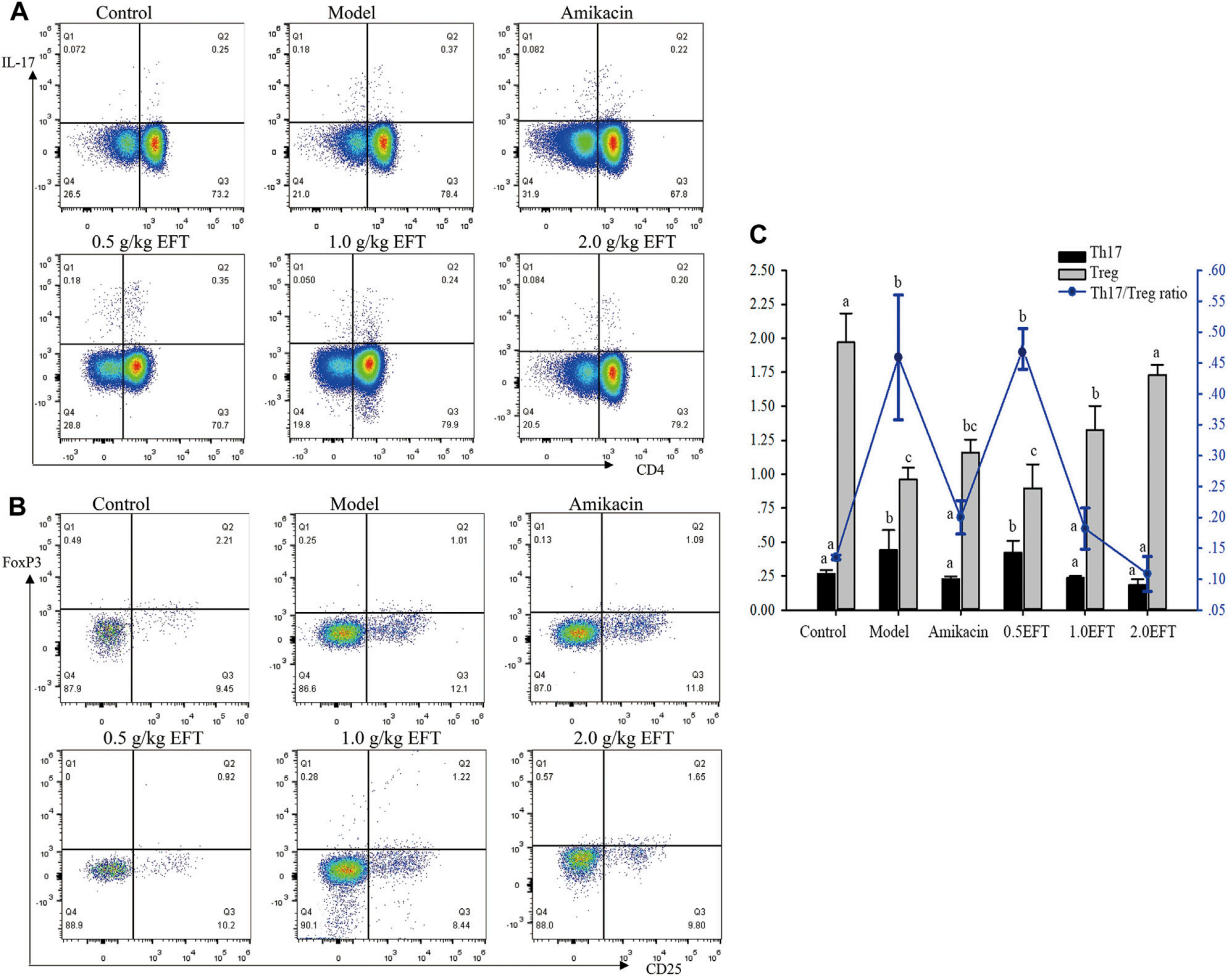
The frequency Treg (CD4+Foxp3+) cells and Th17 (CD4+IL-17+) cells were analyzed by low cytometry. **(A)** Representative pictures for low cytometry of Treg cells, **(B)** representative pictures for low cytometry of Th17 cells, and **(C)** the averages of frequency of Treg and Th17 cells and ratio of Th17/Treg. All values are expressed as mean ± SD (*n* = 6). Different English letters in the same column represented a significant difference through pair-wise comparison by the LSD multiple comparison test (*p <* 0.05).

To verify that the suppressive effects of *T. hemsleyanum* on pneumonia were related to the immune system, the frequencies of splenic Treg cells and Th17 cells were detected by flow cytometry. The results demonstrated that the percentages of Treg cells in the model group were dramatically lower than percentages of those in the sham group (Figure 7), whereas the proportion of Treg in EFT (1 g/kg, 2 g/kg) groups was obviously increased by 37 and 80.21%, respectively, compared with the model group (*p* < 0.05). On the contrary, the numbers of Th17 cell in the model group showed a significant uptrend compared with the control group. Dramatically, the increasing trend was significantly reversed by 46.62 and 57.90% in EFT (1.0 g/kg, 2.0 g/kg) groups, respectively. Ultimately, the ratio of Th17/Treg in EFT groups (1.0 g/kg, 2.0 g/kg) was declined by 60.58 and 76.32% compared with the model group (*p* < 0.05), respectively.

### The Effect of EFT on the mRNA Expressions of RORγt and Foxp3 in the Lungs

**FIGURE 8:**
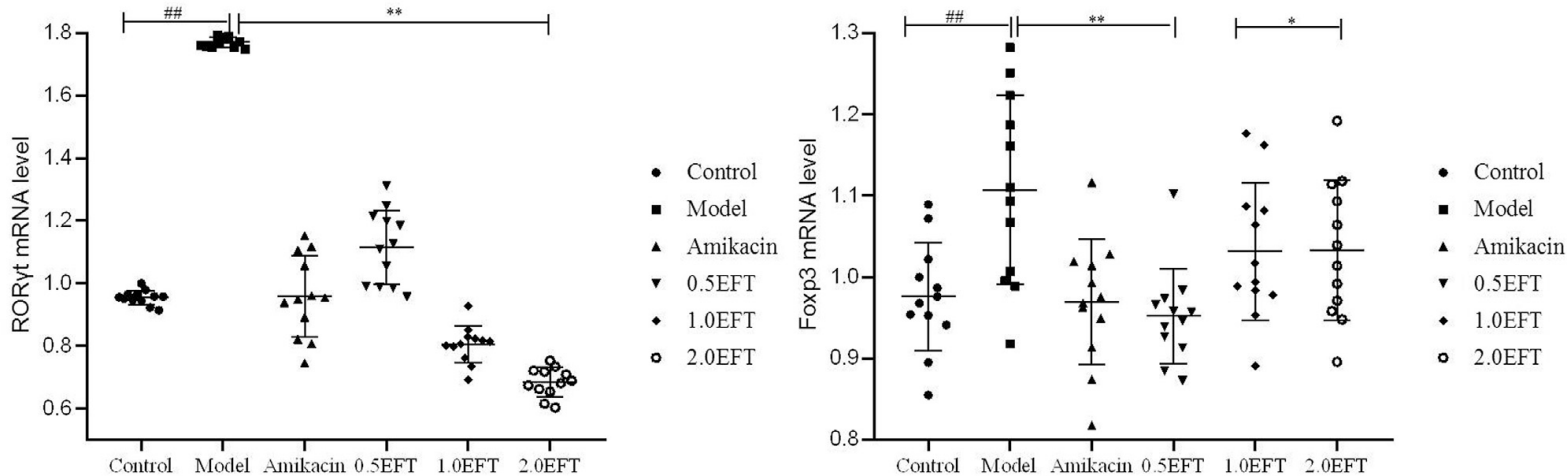
The mRNA expression levels of ROR-γt and Foxp3 in lung tissue. All values are expressed as mean ± SD (*n* = 12). #*p* < 0.05; ##*p* < 0.01 compared with the control group, *p < 0.05; **p < 0.01 compared with the model group.

As the vital transcription factors participated in the development and function of CD4^+^, IL-17^+^ (Th17), CD8^+^, and IL-17^+^ (Tc17) cells, the mRNA expressions of retinoid-related orphan nuclear receptor-γt (RORγt) and forkhead box protein3 (Foxp3) were detected using the RT-PCR method. As shown in Figure 8, no obvious differences in the Foxp3 level were observed between EFT groups and control. The relative content of RORγt in pulmonic tissue of *P. aeruginosa*–infected mice showed a noteworthy upregulation tendency compared with sham control (Figure 8). Treatment with EFT (0.5, 1.0, and 2.0 g/kg) could reduce the RORγt mRNA levels by 37.08, 54.46, and 61.82% (*p* < 0.05), respectively, which revealed that EFT had a dose-dependent inhibition effect on the expression of RORγt.

## Discussion

*P. aeruginosa* lung infection is a typical manifestation of persistent pulmonary diseases, such as cystic fibrosis and chronic obstructive pulmonary disease, characterized by pulmonary inflammation, high levels of cytokine and chemokine production, and massive recruitment of neutrophils. Currently, there are no specific drugs for use in the treatment of *P. aeruginosa* lung infection due to the lack of novel classes of antibiotics as well as the constant increase of multidrug resistance, and most available treatments are symptomatic and supportive. With valuable experience for clinical practice and basic medical research, the application of TCM has become increasingly popular, thanks to its outstanding advantages such as more targets and fewer side effects. Previous studies have shown that TCM not only targeted the etiology of *P. aeruginosa* lung infection but also effectively alleviated a series of complications caused by *P. aeruginosa* lung infection (Chakotiya et al., 2016; Kong et al., 2019; Wang et al., 2020).

*T. hemsleyanum*, a rare perennial and medicinal plant, has been proven to exhibit significant anti-inflammatory (Wang et al., 2018; Chu et al., 2019) and immunomodulatory activities (Ding et al., 2008; Xu et al., 2008) in various animal models. Moreover, available pharmacological studies have indicated that its anti-inflammatory effect was closely associated with inhibiting various inflammatory cytokines or mediators through inhibiting p38MAPK and NF-κB pathways (Liu et al., 2015). Meanwhile, Liu discovered that *T. hemsleyanum* could improve LPS-induced acute lung injury through the TLR4/MD-2–mediated pathway (Liu et al., 2016). Another study demonstrated that chronic obstructive pulmonary rats treated with *T. hemsleyanum* appeared to reduce inflammatory response by decreasing the levels of IL-8 and CRP in both serum and BALF (Wang et al., 2016). Besides, *T. hemsleyanum* has been shown to regulate mice immune functions and modulate Tregs in a mouse tumor model (Feng et al., 2014). However, the therapeutic effect of *T. hemsleyanum* leaf on *P. aeruginosa*–induced pneumonia model has not been reported, and the functional components and the molecular mechanisms were poorly defined.

Recently, network pharmacology provides a whole new way of thinking for identifying multiple components and investigating the mechanisms of TCM. In the present study, in consideration of the complexity of the bioactive constituents in *T. hemsleyanum* leaves and the diversity of potential regulatory targets in humans, network pharmacology analysis was used to predict the target proteins of the compounds from multiple databases and the potential against pneumonia mechanism. In this study, a total of 34 bioactive constituents in the leaves of *T. hemsleyanum*, 124 compound-related targets, and 4,779 pneumonia-related targets were screened from the public databases. Among these targets, 80 targets were cross-targets of compounds and pneumonia, implicating the possible anti-pneumonia action of leaves of *T. hemsleyanum*. They were prominently enriched in several pathways related to pneumonia such as the PI3K–Akt signaling pathway, Ras signaling pathway, ErbB signaling pathway, JAK–STAT signaling pathway, TNF signaling pathway, and AMPK signaling pathway. GO enrichment analysis indicated 80 drug–disease crossover proteins were mainly involved in signal transduction, inflammatory response, and the oxidation–reduction process, which provided a basis for further research.

PI3K–AKT, Ras–MAPK, and JAK–STAT are universally expressed intracellular signal transduction pathways, which participated in both the inflammatory and immune responses (Canttey, 2002; Liu et al., 2020). Moreover, research had proved that Qingfei Xiaoyan Wan ameliorated *P. aeruginosa*–induced acute lung inflammation by regulating PI3K/AKT and Ras/MAPK pathways (Hou et al., 2016). To evaluate and verify the effect of EFT on *P. aeruginosa* lung infection, a mouse model induced by *P. aeruginosa* was established, and the dosage range of EFT was determined on the basis of the clinical dosage and the results of preliminary experimental results. The bacterial burden in the model group was significantly higher than that in the sham group, which was in accordance with the change of histopathology. The pro-inflammatory levels of IL-17, IL-6, and TNF-α in serum and BALFs of model mice showed a significant upward trend, which was consistent with that in the previous report (Song et al., 2003). Interestingly, the levels of anti-inflammatory cytokines manifested the contrary tendency; that is, the expressions of TGF-β1 and IL-10 cytokines were downregulated in BALFs, and both displayed an unexpected upward trend in serum, but there was no significant difference between the groups. Noticeably, EFT treatment reduced the levels of pro-inflammatory factors and increased the anti-inflammatory levels. As for the level of TGF-β1 and IL-10 in serum, the EFT (2 g/kg) group showed an increasing trend compared with the EFT (1 g/kg) group or EFT (0.5 g/kg).

It has been recognized that T lymphocytes, especially CD4^+^ T cells, played a crucial part in the progression of autoimmune and inflammatory diseases. Th17 cells, whose development relied on the transcription factor RORγt, are one of CD4^+^ T effecter cell subsets. Th17 cells are regarded as playing a role in immune inflammatory diseases characterized by the secretion of IL-17A and other cytokines, such as IL-6, IL-21, and IL-22 (Paquissi, 2007). Although the function of Th17 cells has been studied in several lung diseases, such as asthma (Newcomb and Peebles, 2013), tuberculosis (Kononova et al., 2014), lung cancer (Chang et al., 2014), and chronic obstructive pulmonary disease (Vargas-Rojas et al., 2011), there were few data about it in *P. aeruginosa* lung infection patients. The results of the present study showed that the frequency of Th17 cells, the levels of pro-inflammatory cytokines (IL-17, IL-6, and TNF-α), and the expression of the transcription factor RORγt were conspicuously increased in the model group, compared with the control group. EFT could significantly reverse those increasing tendency, similar to the positive drug amikacin. Moreover, there was a positive correlation between the reversal effect and the concentration of EFT.

Treg/Th17 cells kept a dynamic balance in normal physiological conditions. Once the balance between Th17 and Treg cells is broken, it may lead to a series of immune inflammatory diseases and suppression of the immune response. Clinical studies have shown that the balance between Th17 and Treg played a vital role in the pathogenesis of allergic airway inflammation (Gao et al., 2017). The Th17/Treg ratio of rats with smoke inhalation–induced acute lung injury (ALI) was positively correlated with the lung injury score (Zhang et al., 2016). In the current study, the IL-10 level of anti-inflammatory cytokine was decreased in the model group, compared with the control group; high dose (2.0 g/kg) of EFT treatment groups manifested a notable increase of the IL-10 level. EFT treatment had no significant effect on Foxp3 expression. Then we examined the change in the Th17/Treg ratio in mice lungs and the expression of the transcription factor Foxp3. The Th17/Treg ratio of the model group increased significantly but that of medium and high dose of EFT treatment groups decreased to the control level; the lowering effect was comparable to that of the positive drug amikacin group. These results suggested that the anti-inflammatory properties of *T. hemsleyanum* might also be attributed to its ability of maintaining Treg/Th17 balance in activated lymphocytes and suppressing the expression of pro-inflammatory cytokines.

## Conclusion

In summary, our study explored drug–disease target interactions and multi-target mechanism of *T. hemsleyanum* leaves on pneumonia using the network pharmacology approach, simultaneously verifying the curative effect of *T. hemsleyanum* leaf on the lung injury *in vivo*. The results of animal experiments have shown for the first time that EFT might contribute to relieve pneumonia induced by *P. aeruginosa* treatment, mainly through regulating the Treg/Th17 cell balance and suppressing the expression of pro-inflammatory cytokine levels. This new finding not only extended our understanding of the underlying mechanisms of the anti-inflammatory activity of *T. hemsleyanum* on the cellular but also laid the foundation for the future application of this herb in regulating T-cell homeostasis that contributed to immune-mediated diseases.

However, there were some limitations which still require further evaluation and exploration. First, due to the characteristics of network pharmacology, the results of network pharmacology analysis in this article depended on the discovery and mining of more chemical components. Second, multiple signaling pathways were identified through network pharmacological analysis; we only selected inflammatory and immune factors to further analyze and confirm the mechanism of *P. aeruginosa* lung infection *via in vivo* experiment. Third, the protective mechanism of EFT against *P. aeruginosa* lung infection needed more deeply experimental confirmation.

## Data Availability

Publicly available databases were analyzed in our study. The targets of the compounds of *T. hemsleyanum* can be found in Swiss Target Prediction database (http://www.swisstargetprediction.ch/) and UniProt database (https://www.uniprot.org/). The putative target genes of pneumonia were from DigSee (http://210.107.182.61/geneSearch/), GeneCards (https://www.genecards.org/) and OMIM (http://www.ncbi.nlm.nih.gov/omim) databases. The protein-protein interactions (PPI) of each target were generated by String database (http://string-db.org/). Enrichment analysis were performed using DAVID (https://david.ncifcrf.gov/). Complementary data are supplied as Supplementary Material.

## Abbreviations

BALFs, bronchoalveolar lavage fluids; CFU, colony-forming unit; EFT, aqueous extract of *T. hemsleyanum* leaf; ELISA, enzyme-linked immunosorbent assay; Foxp3, forkhead box protein 3; GAPDH, glyceraldehyde-3-phosphate dehydrogenase; HAP, hospital-acquired pneumonia; HE, hematoxylin–eosin; IL-17, interleukin-17; *P. aeruginosa*, *Pseudomonas aeruginosa*; RORγt, retinoid-related orphan nuclear receptor-γt; TCM, traditional Chinese medicine; TGF-β1, transforming growth factor-β1; TNF-α, tumor necrosis factor-α.
