# Supplementary material for: Extract From Tetrastigma hemsleyanum Leaf Alleviates Pseudomonas aeruginosa Lung Infection: Network Pharmacology Analysis and Experimental Evidence
Source: Front Pharmacol. 2021 Jul 19;12:587850. doi: 10.3389/fphar.2021.587850 (PMC8326761; doi:10.3389/fphar.2021.587850)
Supplement: Supplementary file 1 [file DataSheet1.PDF]

Supplementary Material

1.1 Supplementary Figures

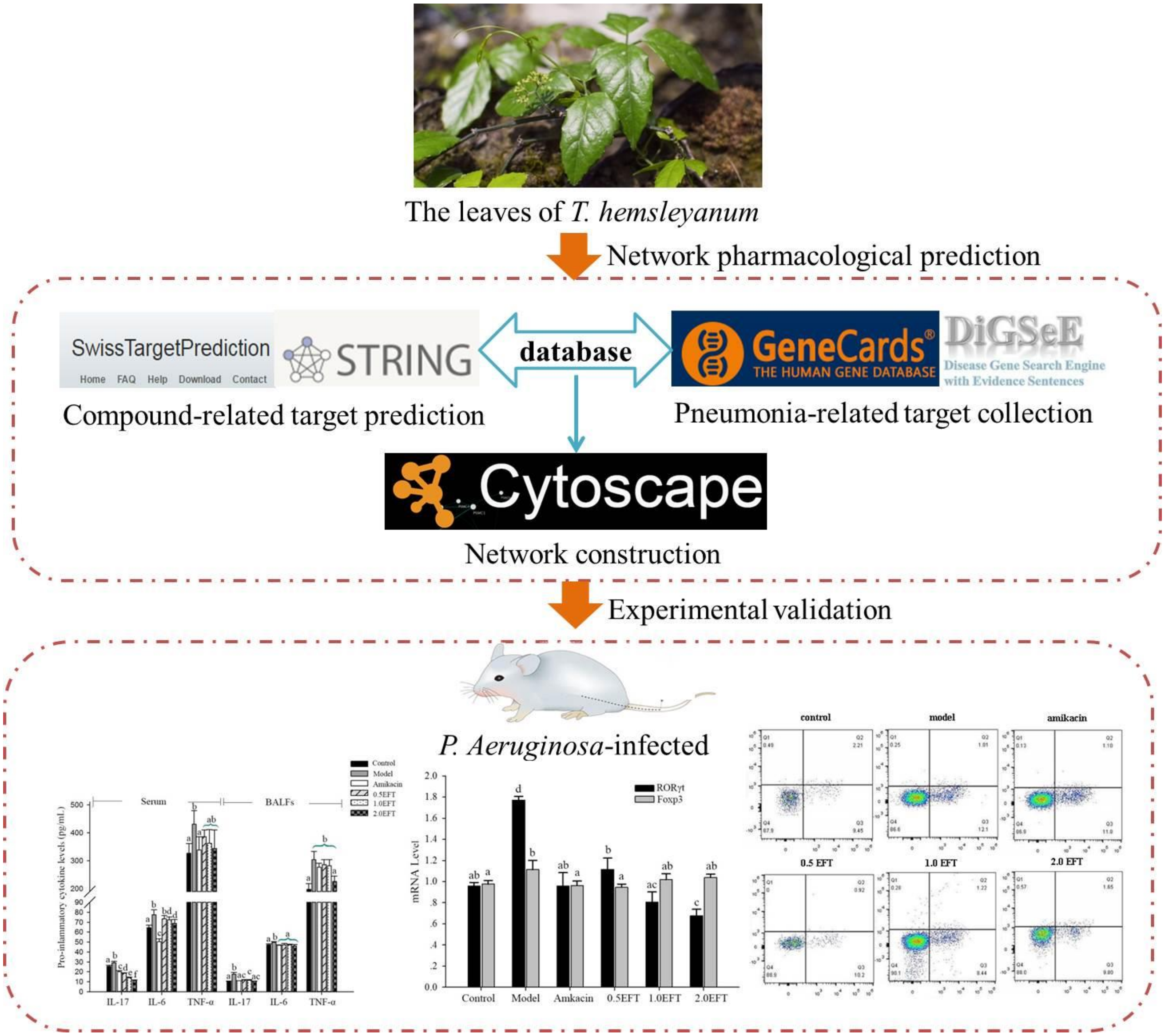

Supplementary Figure 1. Flowchart of the network pharmacology approach for deciphering the therapeutic mechanisms of action of *Tetrastigma hemsleyanum* leaf on *Pseudomonas aeruginosa* lung infection

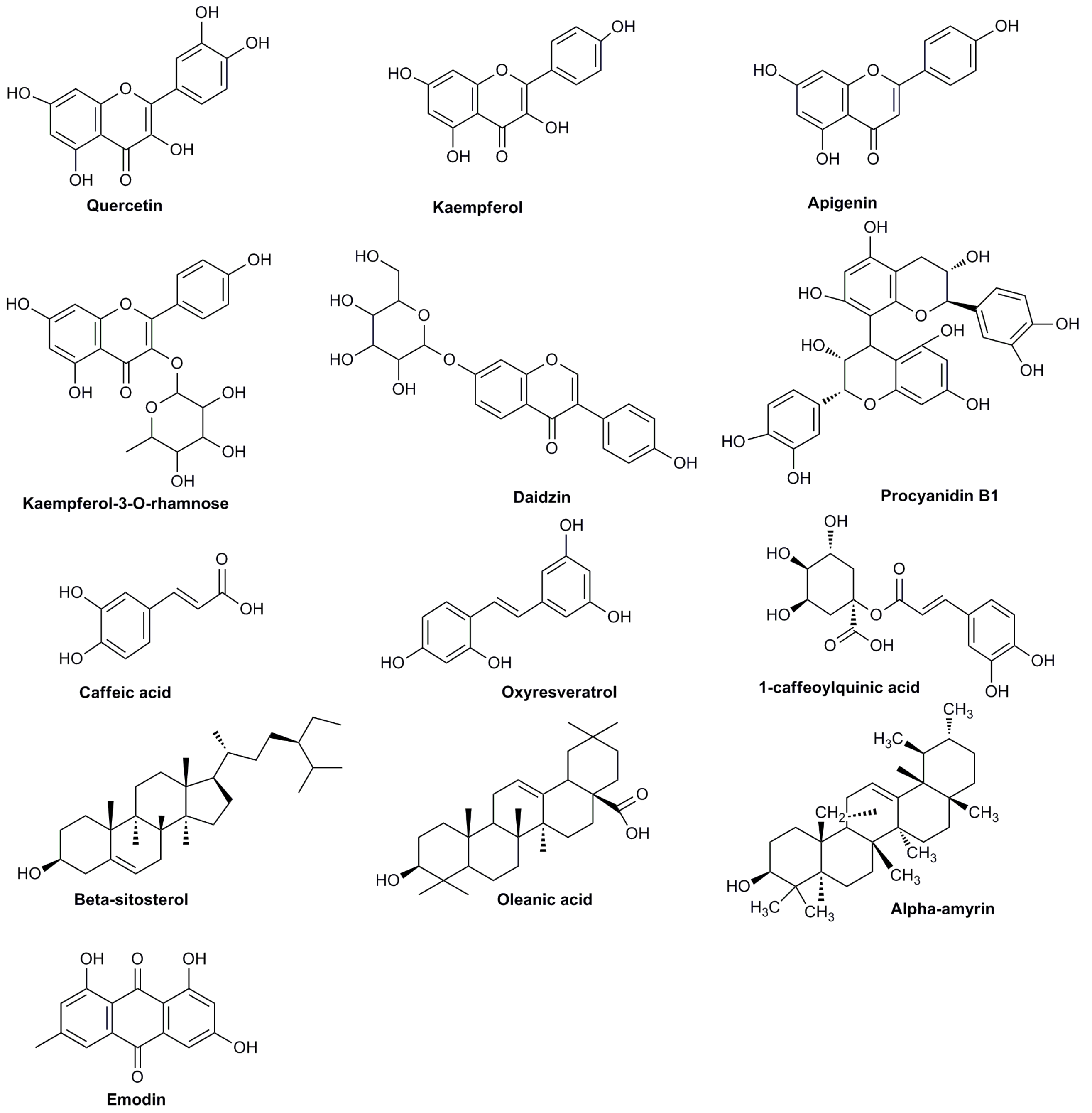

Supplementary Figure 2. Structures of 13 bioactive compounds isolated from *T. hemsleyanum* leaf in the treatment of pneumonia based on network pharmacology

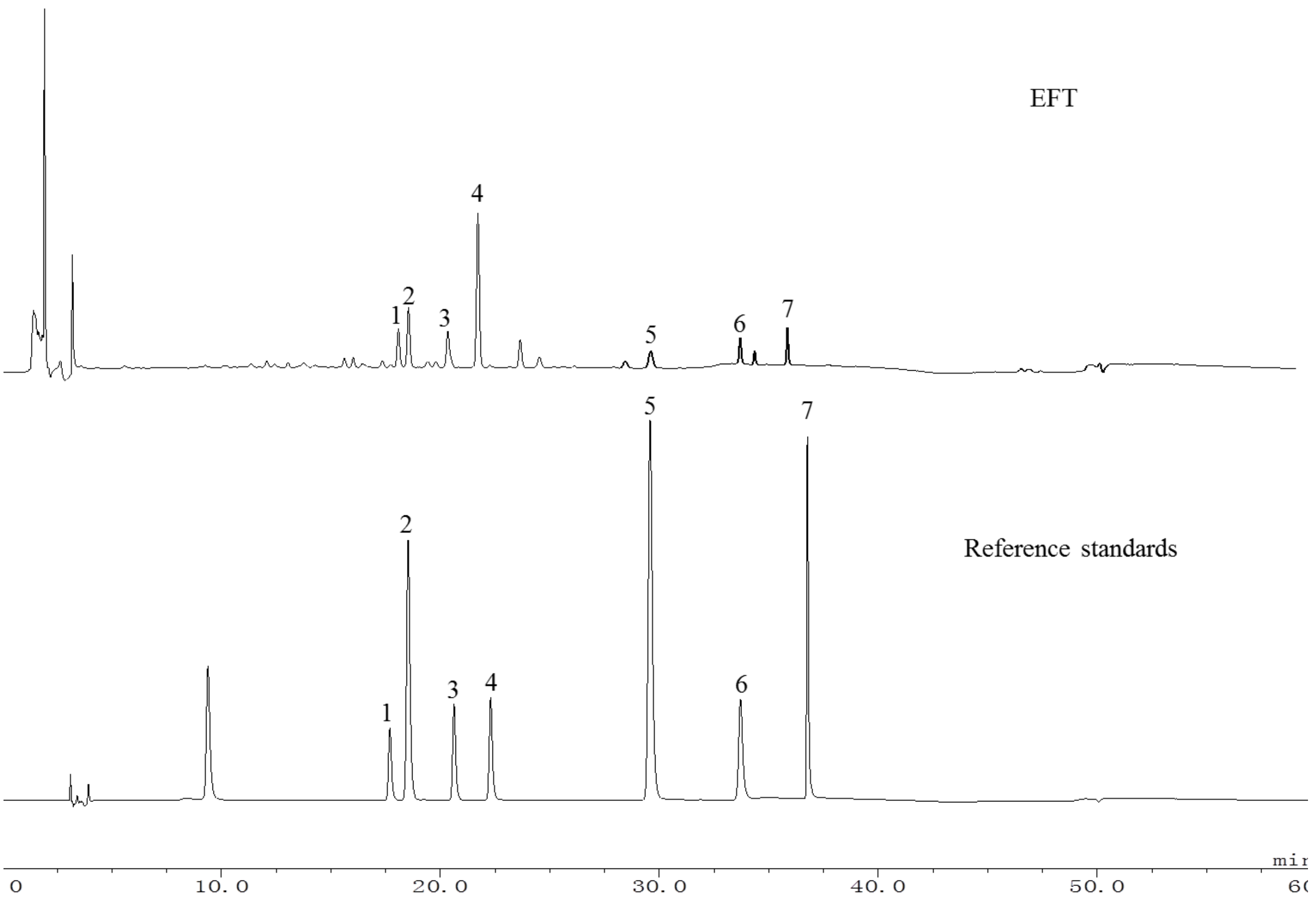

Supplementary Figure 3. The HPLC chromatograms of reference standards and EFT sample. 1. quercetin-3-O-rutinoside; 2. β-sitosterol; 3. kaempferol-3-O-rutinoside; 4. kaempferol-3-glucoside; 5. resveratrol; 6. quercetin; 7. kaempferol
